# Differences across herds with different dairy breeds in daily milk yield based proxies for resilience

**DOI:** 10.3389/fgene.2023.1120073

**Published:** 2023-06-01

**Authors:** Ines Adriaens, Gerbrich Bonekamp, Jan Ten Napel, Claudia Kamphuis, Yvette De Haas

**Affiliations:** ^1^Animal Breeding and Genomics, Wageningen Livestock Research, Wageningen University and Research, Wageningen, Netherlands; ^2^Department of Biosystems, Livestock Technology, KU Leuven, Leuven, Belgium

**Keywords:** resilience, precision phenotyping, dairy cow, breed differences, herd differences, robustness

## Abstract

Global sustainability issues such as climate change, biodiversity loss and food security require food systems to become more resource efficient and better embedded in the local environment. This needs a transition towards more diverse, circular and low-input dairy farming systems with animals best suited to the specific environmental conditions. When varying environmental challenges are posed to animals, cows need to become resilient to disturbances they face. This resilience of dairy cows for disturbances can be quantified using sensor features and resilience indicators derived from daily milk yield records. The aim of this study was to explore milk yield based sensor features and resilience indicators for different cattle groups according to their breeds and herds. To this end, we calculated 40 different features to describe the dynamics and variability in milk production of first parity dairy cows. After correction for milk production level, we found that various aspects of the milk yield dynamics, milk yield variability and perturbation characteristics indeed differed across herds and breeds. On farms with a lower breed proportion of Holstein Friesian across cows, there was more variability in the milk yield, but perturbations were less severe upon critical disturbances. Non-Holstein Friesian breeds had a more stable milk production with less (severe) perturbations. These differences can be attributed to differences in genetics, environments, or both. This study demonstrates the potential to use milk yield sensor features and resilience indicators as a tool to quantify how cows cope with more dynamic production conditions and select animals for features that best suit a farms’ breeding goal and specific environment.

## 1 Introduction

Global sustainability issues such as climate change, biodiversity loss and food security require food systems to become more resource efficient and better embedded in the local environment. For this reason, it is generally believed that an agricultural transition towards more diverse, circular and low-input dairy farming with animals best suited to the specific production situation is needed. This type of dairy farming can be a resource-efficient way of producing high quality food for human consumption (van Hal et al., 2019). The design and optimization of these non-intensive dairy farming systems largely depends on local circumstances, but they have in common that mainly grass and rest products from other industries are used as animal feed (van Hal et al., 2019). In less intensive dairy farming systems, cows might be exposed to more environmental disturbances such as variable feed quality or weather extremes when they are kept outdoors. Understanding to what extent cows are affected by these environmental disturbances is crucial to improve animal functioning and welfare in extensive farming systems. More specifically, the cows should be minimally affected by a disturbance or rapidly recover after exposure, generally accepted as being “resilient” (Colditz and Hine, 2016; Berghof et al., 2019) or “robust” (Ten Napel et al., 2009).

Farmers aim to maintain health, fertility, longevity and production of cows at an acceptable level. One strategy is to avoid exposure to any disturbances as much as possible by controlling the production environment (Control Model; Ten Napel et al., 2006). Breeds selected in such a regime tend to depend more heavily on favourable conditions (so called ‘specialists’; Bryant et al., 2006; Parsons, 1997). Another strategy is to minimise the impact in the presence of disturbances (Adaptation Model; Ten Napel et al., 2006). Developing and utilizing resilience of cows is particularly relevant in the latter case. Breeds selected in more diverse and dynamic conditions are referred to as ‘generalists’ (Parsons, 1997; Bryant et al., 2006).

Traits for which resilience is relevant such as health, fertility, longevity and production level, differ between cattle breeds. For instance, Bieber et al. (2019) showed that local breeds from Sweden, Austria, Switzerland and Poland tend to live longer, have a better health and fertility, but produce less than breeds such as Holstein Friesian (HOL) or Brown Swiss (BSW) which are globally used in intensive dairy farming systems. Bieber and others described the local breeds to be more robust and, therefore, more suitable for locally adapted organic or agroecological dairy farming (Bieber et al., 2019).

Resilience of dairy cows may be improved via genetic selection or farm management. For both methods, large-scale quantification of this trait is required (Berghof et al., 2019). Scheffer et al. (2018) proposed to quantify resilience using automated high-frequency records of traits that could be affected by disturbances, for example, milk yield in dairy cattle (Elgersma et al., 2018). Therefore, it was proposed in previous work to use mathematical features of fluctuations in daily milk yield (DMY) to quantify the level of resilience of individual cows (Colditz and Hine, 2016; Elgersma et al., 2018; Adriaens et al., 2020; Poppe et al., 2020). The main limitation of this approach is that it only quantifies the ‘milk yield’ resilience, and it is not a direct measure of health or longevity. Still, it was demonstrated to be a useful tool. For example, Poppe et al. (2020) derived resilience indicators from the daily deviations of DMY records from an expected lactation curve to be used for genetic selection. Using a dataset consisting of purebred HOL cows only, they found that a low variance and autocorrelation of daily deviations was correlated with fewer health problems, suggesting these cows had a high level of resilience. In another study that focussed on farm management, mathematical features from DMY records were used to create predictive models for the “lifetime resilience rank” of animals in a herd (Adriaens et al., 2020; Ouweltjes et al., 2021). This rank takes lifetime longevity, health, fertility and production traits into account to quantify long-term consequences of cows’ resilience (Friggens et al., 2022). These previous studies demonstrated the potential of comparing how cows are affected by disturbances based on mathematical features calculated from first parity DMY curves. A main finding in this regard is that, besides the level of milk yield resilience of individual cows, also management and culling strategies strongly affect the DMY dynamics, and that therefore differences in farm management should be taken into account when studying resilience of dairy cattle.

To date, large scale analyses of resilience traits based on DMY have been performed on HOL cows only, while in the context of diversification of the dairy sector, it is crucial to understand and quantify resilience also for breeds other than HOL, or for crossbred animals. To this end, it is useful to explore the sensor features and resilience indicators calculated on a more diverse set of data including non-HOL breeds and animals with mixed genetics. Unfortunately, a classical breed comparison for these features is currently impossible, as it would require explicitly considering the herd and environment effect. For this, insufficient data are available from farms in which multiple animals of different breeds are kept in the same environment to reach sufficient statistical power to draw a conclusion. The aim of this study was to compare sensor features as proposed by Adriaens et al. (2020) and resilience indicators as defined by Poppe et al. (2020) 1) across breeds and cross-bred animals, and 2) between herds with a low or high number of HOL cows. This will indicate which sensor features and resilience indicators can be used to differentiate between herds grouped by non-HOL breed and herds using HOL almost exclusively. This is needed to gain insight in to what extent high-frequency data can help phenotyping complex traits and to formulate proper recommendations towards a more sustainable and diverse dairy sector.

## 2 Materials and methods

### 2.1 Data selection and preparation

A dataset of 4,053 herds and 535,104 first parity lactation curves with high-frequency DMY data was obtained from breeding organisation CRV (Arnhem, the Netherlands). All DMY data originated from farms with automated milk yield systems, but details on the brand, type or specifics of these robots were not available due to privacy and GDPR regulations. Still, as these data are typically recorded by sensors approved by ICAR ensuring similar accuracy and reliability, and the same data collection protocol is used to collect the data from the farms, they can be considered comparable over farms. Besides the first parity milk yield, the dataset contained information on herd characteristics (e.g., herd size, date of last milking available) and animal identification and ancillary information (birth and calving dates, age at first calving, breed information). Several data editing steps were applied to select lactation curves with a sufficient amount and quality of milk yield records. First, the cows that calved for the first time before 2010 were excluded. For the remaining animals, the first lactation data was summarized. Only lactations for which at least 100 days of data, starting within 5 days after calving and with at most 5 successive days of data missing were retained, similar to the criteria as used by Poppe et al. (2020) utilizing the same dataset. Next, the two subsets for the analysis were constructed. Subset A contained data from cows selected based on breed criteria (Section 2.1.1). Subset B contained data of cows from herds selected based on the average percentage of HOL across cows in the herd (Section 2.1.2).

The initial selection steps were performed with a bash script run on the high performance computer of Wageningen University and Research, using the AWK programming language (Aho et al., 1988) and R (version 3.2.2; R Project for Statistical Computing, Vienna, Austria). Lactation selection, data preparation and calculation of the sensor features and resilience indicators (Section 2.2) was done using Matlab R2020b (The Mathworks Inc., Natick, MA, United States). The statistical analysis (Section 2.3) was implemented with Python 3.9, using the software packages “pandas-v1.4.2”, “numpy-v1.22.3”, “statsmodels-v0.13.2”, and “matplotlib-v3.5.1” for the visualisations.

#### 2.1.1 Subset A-selection based on breed criteria

For the comparison of sensor features and resilience indicators across purebred animals of different breeds and HOL crossbred animals given their specific production environment, we selected the purebreds (at least 87.5% of the genes of a single breed) and crossbreds (50% HOL and 50% of another known breed) of the dataset. The resulting data were highly imbalanced towards purebred HOL cows which might impact the statistical analysis. Therefore, a representative sample of 22,100 HOL cows, equal to the total number of animals available for the other breeds and crossbreds, was selected for inclusion in the analysis. To this end, we sorted the HOL cows (n = 395,654) successively for 1) age at first calving; 2) average DMY; 3) calf date; and 4) maximal number of herd mates. After each sorting step, a uniform sample of 5,525 animals was taken by selecting one animal every n/5,525 animals, and removing these animals for the selection in the next sorting step. This way, the entire population range for the different traits of interest is included in the analysis and selection of related cows is avoided. This selection was rather mild and still captures the variability present within the HOL breed, while bringing its size to an acceptable order of magnitude for the statistical analysis. Breeds that were insufficiently represented in the dataset (less than 10 unique crossbred and purebred animals combined) were removed. This resulted in a dataset (Subset A) that consisted of data from 39,643 cows from in total 3,393 herds. From these, 22,911 cows were purebred animals originating from 3,184 unique herds. The remaining 16,732 crossbred cows originated from 1,770 herds. The most prevalent crosses in our dataset were those with Simmental Fleckvieh (SIM) animals (*n* = 6,367), followed by Montbéliarde (MON) (*n* = 3,002), BSW (*n* = 2,622), Swedish Red (SRB) (*n* = 1,882) and Norwegian Red (NRF) (*n* = 1,264). A full overview of the number of animals and herds per breed and per crossbred combination, together with their average DMY, uncorrected for herd effects, is given in Table 1. In summary, the number of purebred animals included in the analysis ranged from 11 Groninger Whiteheaded **(GRO)** to 19,740 (HOL). Some breeds had no purebred animals and were only included via crossbreds: NRF, SRB, Ayrshire (AYR) and Belgian Blue (BBL).

**TABLE 1:** Overview of the cows in Subset A selected for breed criteria. The data below the cross are the crossbred animals, for which 4/8th is Holstein Friesian (HOL), and the remaining part the indicated breed. No correction for the herd-environment effect was applied, and therefore the breed differences in daily milk yield (DMY) need to be interpreted as the combined effect of breed and farm environment.

Breed^a^	No. cows	No. herds	DMY^b^	
			Mean	Std.
HOL	19,740	3,087	27.2	5.2
MRY	1,320	58	20.4	3.8
JER	665	32	18.1	3.8
MON	432	16	20.4	4.4
SIM	397	48	22.3	5.1
DFR	256	8	21	3.1
BSW	90	12	21.8	4.2
GRO	11	3	14.9	4.4
Cross	16,732	1,770	25.4	5.1
MRY	761	250	23.4	4.6
JER	326	92	23	4.3
MON	3,002	396	26.7	5.1
SIM	6,267	782	25.5	5.2
DFR	51	27	24.5	4.1
BSW	2,622	582	25.2	5
GRO	127	74	21.4	4.8
SRB	1,882	380	24.4	4.7
NRF	1,264	304	26.3	4.6
BBL	393	286	24.7	5.7
AYR	37	20	24.1	4.5

^a^ HOL, holstein friesian; MRY, Meuse-Rhine-Yssel, JER, jersey; MON, Montbéliarde; SIM, simmental fleckvieh; DFR, dutch friesian; BSW, brown swiss; GRO, groninger whiteheaded, cross = crossbred animals, SRB, swedish red; NRF, norwegian red; BBL, belgian blue; AYR, Ayrshire.

^b^ DMY, daily milk yield.

Breed and herd effects cannot be distinguished in these data. Still, it enables quantifying the sensor features and resilience indicators for the available animals and available differences across the breeds (i.e., breed effects) can be interpreted as the combined result of breed and environment.

#### 2.1.2 Subset B- selection based on herd criteria

The second subset served the comparison of sensor features and resilience indicators across herds with either very high (above 99%, SEL99) or low (below 50%, SEL50) percentages of HOL genetics in the herd. For this, we selected herds based on the average proportion of HOL across cows in the herd. We included all cows of the selected herds. First, herds with a herd size of less than 50 or more than 300 animals were discarded. Next, we selected all the herds with less than 50% HOL (*n* = 181). A representative subset of the herds with 99% or more HOL (*n* = 406) was selected with a similar procedure as described in Section 2.1.1. More specifically, the 406 farms were consecutively sorted for 1) average age at first calving, 2) average DMY, 3) total number of animals in the herd, and 4) maximal number of animals in the herd in a single year. After each sorting step, a uniform sample of 45 herds was selected, resulting in a representative sample of in total 180 herds.

### 2.2 Calculation of sensor features and resilience indicators

#### 2.2.1 Milk yield modelling

For each cow in the dataset, we quantified the first parity DMY dynamics. To this end, two models were fitted on the individual lactation curves: 1) a Wood model to describe the lactation dynamics *as is* on all the data regardless of perturbations, and 2) a 4th order quantile regression curve to estimate the ‘expected’ lactation curve, i.e., the production dynamics in the situation of no perturbations present. The Wood model has 3 parameters (Wood, 1967) and is calculated as follows: *DMY* = a*DIM^b^*e^-c*DIM^ with *DMY* the daily milk yield, *DIM* = days in milk and *a, b* and *c* the parameters determining the shape of the lactation curve. The quantile regression model represents the lactation dynamics in absence of milk yield perturbations caused by, e.g., health or other environmental disturbances. For this, we used a fourth order polynomial quantile regression as proposed by Poppe et al. (2020), fitted on the DMY from day 11–340 after calving with a conditional quantile of 0.7.

#### 2.2.2 Sensor features and resilience indicators

Milk yield based sensor features as defined by Adriaens et al. (2020) and resilience indicators as proposed by Poppe et al. (2020), 40 in total, were calculated based on the first parity DMY and the abovementioned milk yield models (Section 2.2.1). More specifically, starting from the models’ parameters, we calculated the shape and characteristics of the lactation curves, including the peak and day in milk of peak, persistency, slope to peak of both the fitted Wood model and the expected lactation curve. Furthermore, the residuals of these models were characterised by calculating the variance, autocorrelation, skewness, the maximal and average residual to quantify the DMY variation. Besides the residuals, also the perturbations, defined as deviations from the expected lactation curve were quantified. A complete overview of the sensor features and resilience indicators is given in the Supplementary Material, Supplementary Table S1. We partitioned the 40 sensor features and resilience indicators into six different categories: 1) the dynamics of the first parity lactation curve as calculated with the Wood model, including the slopes before and after the peak, the peak production, peak DIM and accumulated yield in the first 50–305 days; 2) the characteristics of the residuals of the Wood model as fitted on all the data to quantify the lactation shape including perturbations; 3) the dynamics of the expected lactation curve including peak production, slope to peak, days in milk at peak and persistency; 4) the characteristics of the residuals of the expected lactation curve and its goodness-of-fit measures; 5) the number of perturbations, defined as impact of a disturbance, and their characteristics as calculated from the expected lactation curve; and 6) the resilience indicators as proposed by Poppe et al. (2020), i.e., the log transformed variance of the residuals calculated from the expected lactation curve, and the lag-1 autocorrelation.

### 2.3 Statistical analyses

#### 2.3.1 Outlier detection

Outliers in the sensor features and resilience indicators can result both from mathematical or data artefacts and from biological or physiological aberrations. Sensor feature observations with very extreme values were removed based on the Cook’s distance which represents for each observation how much it influences a regression model. Its formula is as follows:

$$D_{i}=\frac{r_{i}^{2}}{p*MSE}*\;\frac{h_{ii}}{{\left(1-h_{ii}\right)}^{2}}$$ (1)

where $r_{i}$ is the *i*th residual, *p* is the number of coefficients in the regression model, MSE the mean squared error calculated as $MSE=\frac{1}{n}*\sum_{i=1}^{n}{\left(y_{i}-\hat{y}\right)}^{2}$ , and $h_{ii}$ the *i*th leverage value of the model that quantifies how much the observation affects the predicted response $\hat{y}$ . The regression model fitted to calculate these Cook’s distances were the same as detailed in Section 2.3.2 and Section 2.3.3. Observations that were in the 99.5^th^ to 100th quantile (the 0.5% most influential observations) were removed from further analysis of each feature, resulting in a removal of approximately 185 datapoints = 0.5% of observations per model.

#### 2.3.2 Subset A—Selection based on breed criteria

The combined effects of breed and environment on the 40 sensor features and resilience indicators were estimated using a linear regression model. Before fitting the model, a min-max standardisation was applied to the sensor features and resilience indicators for better interpretation and scaling of the regression coefficients. Furthermore, a covariate for the scaling effect of average DMY was included as proposed by Bonekamp et al. (2022) and Poppe et al. (2020), because an increase in the mean of a trait typically results in a (proportional) increase of its variance (Falconer and Mackey, 1996). The final model equation was (Eq. 2):

SF=1+SIM+MON+BSW+SRB+NRF+MRY+BBL+JER+GRO+DFR+AYR+cross+aDMY+ε (2)

with *SF* the observed sensor feature or resilience indicator, *aDMY* the min-max standardised average DMY of each cow, and ε the a random error term. The HOL breed was combined with the overall mean and taken as intercept (i.e., “1” in Eq. 2), whereas the other variables represent the individual breed proportions, with respectively *SIM* as Simmental Fleckvieh, *MON* as Montbéliarde, *BSW* as Brown Swiss, *SRB* as Swedish Red, *NRF* as Norwegian Red, *MRY* as Meuse-Rhine-Yssel, *BBL* as Belgian Blue, *JER* as Jersey, GRO as Groninger Whiteheaded, *DFR* as Dutch Friesian and *AYR* the proportion of Ayrshire. Purebred cows had a proportion of “1” for the respective breed variable, whereas crossbred animals had a “0.5” for the breed that was not HOL. “ cross ” was the fixed effect of an animal being a crossbred 1) or purebred (0) cow to estimate the heterosis effect. To determine whether the breed or heterosis had a significant effect on the sensor features as compared with the HOL cows, we set a significance threshold of α = 0.05/*n* = 0.05/40 = 0.00125, considering a Bonferroni correction for accumulation of chance in multiple testing, as this model was fitted for 40 different traits.

#### 2.3.3 Subset B—Selection based on herd criteria

Also for this subset the sensor features and resilience indicators were standardised with a min-max standardisation. The regression model tested whether there was an effect of the selection on the sensor features and resilience indicators, i.e., SEL50 vs. SEL99, thereby considering a potential scaling effect of the average DMY. The model equation was as follows:

SF=1+SEL+aDMY+ε (3)

With *SF* the sensor feature, *SEL* the categorical variable representing the selection to which the farm belongs to (less than 50 or more than 99% HOL) and *aDMY* the min-max standardised covariate for the average DMY of the cow in her first lactation. Also here, a significance threshold of *α* = 0.00125 was used to determine whether SEL had an effect on the sensor features and resilience indicators.

##### 2.3.3.1 Differences across HOL cows in SEL50 and SEL99

Differences in sensor features and resilience indicators can result both from breed effects and differences in management across the farms. We did not have access to information that allows to formally distinguish between both effects. One way to get more insight in this aspect, however, is to compare the sensor features and resilience indicators of the purebred HOL animals of both datasets. We, therefore, selected the purebred HOL cows of each SEL and looked into the differences between their sensor features and resilience indicators with the same model as defined in Eq. 3.

## 3 Results

### 3.1 Breed comparison

#### 3.1.1 Dataset description

An overview of the animals in the dataset is given in Table 1 and can be found in the Materials and Methods section. To give an idea of the average production performance of the purebred and crossbred animals, uncorrected for the herd effects, we included the mean DMY. This varied for the purebreds from 14.9 kg (GRO) to 27.2 kg (HOL). All purebred cows were older at first calving than HOL and crossbred animals (data not shown).

#### 3.1.2 Sensor features and resilience indicators

The statistical analysis highlights that a lot of variation in the sensor features and resilience indicators exists, both within and between the breeds when the environmental variation cannot be quantified. The model coefficients and corresponding *p*-values are given in the Supplementary Material (part II, Supplementary Tables S5, S6, sheet ‘Regression’). More specifically, we found that the individual-animal average DMY has a significant effect in more than 90% of the sensor features and resilience indicators, indicating that a milk yield scaling effect indeed needs to be considered. The *R* ^2^ of the models was generally low, demonstrating that only a small part of the variability is explained by the breed, heterosis or milk yield scaling effect.

To understand which type or category of sensor features (Section 2.2.2) differ most often from HOL, we summarized the model results in Table 2. This table provides an overview of the percentage of sensor features and resilience indicators that differ significantly from those of HOL cows, for each breed separately. More specifically, when, for example, the breed-environment effect for 8 out of 11 features based on the residuals of the expected lactation curve differed from HOL, this will be represented in the table as (8/11)*100 = 72.7%. Below, we discuss these findings in more detail.

**TABLE 2:** The percentages breed-environment effects on sensor features and resilience indicators that are significantly different from (HOL) purebred animals. The categories and specific sensor feature descriptions are given in Supplementary Table S1 of the Supplementary Material.

Breed^a^	Wood curve	Wood residuals	Expected curve	Expected residuals	Perturbations	Resilience indicators	Total
Category	1	2	3	4	5	6	
No. of features	6	6	4	11	11	2	40
SIM	66.7	66.7	25.0	81.8	27.3	50.0	55.0
MON	83.3	33.3	50.0	18.2	54.5	50.0	45.0
BSW	66.7	0.0	75.0	36.4	9.1	50.0	32.5
SRB	66.7	16.7	50.0	9.1	0.0	50.0	22.5
NRF	0.0	50.0	25.0	54.5	45.5	100.0	42.5
MRY	83.3	83.3	75.0	63.6	72.7	50.0	72.5
BBL	33.3	33.3	75.0	72.7	81.8	0.0	60.0
JER	100.0	66.7	75.0	63.6	90.9	50.0	77.5
GRO	0.0	33.3	0.0	54.5	45.5	50.0	35.0
DFR	33.3	66.7	100.0	63.6	54.5	100.0	62.5
AYR	0.0	0.0	0.0	0.0	0.0	0.0	0.0
Cross	33.3	33.3	50.0	0.0	9.1	0.0	17.5

^a^ Breeds: SIM, simmental fleckvieh; MON, Montbéliarde; BSW, brown swiss; SRB, swedish red; NRF, norwegian red; MRY, Meuse-Rhine-Yssel, BBL, belgian blue; JER, jersey; GRO, groninger whiteheaded; DFR, dutch friesian; AYR, ayrshire, cross = crossbreds with Holstein Friesian (HOL).

For only 17.5% of the sensor features and resilience indicators a significant heterosis effect was found. This indicates either that the additional effect of a cow being crossbred seems to have no effect on the sensor features and resilience indicators or that differences in environments between pure and crossbred animals masks the effect. Features that describe the shape and dynamics of the Wood curve and the expected lactation curve (category 1 and 3, i.e., peak production, days in milk of peak, persistency, etc.), and thus, quantify the production rather than resilience, differed significantly for most breeds when compared with HOL. More specifically, the positive or negative sign of the estimates consistently suggests that the non-HOL cows have a lower peak production at earlier DIM, and a less steep slope towards the peak, which is consistent with what is expected for cows with a lower DMY.

Category 2 (Table 2) contains the 6 sensor features calculated from the residuals of the Wood curve. We found that these features for the BSW, MON and SRB cows do generally not differ from the HOL. This suggests that the variability in milk production of these breeds is equally high as HOL cows. When the production environment is similar to the one of HOL cows, it suggests that they are equally affected by these disturbances. When their ‘average’ environment, however, differs from the average environment of the HOL, it depends on the actual amount and severity of disturbances whether the variability in milk production is lower or higher than the HOL. The first is expected to be the case when the animals are kept in environments with more disturbances, whereas the latter is true when the cows are in herds with less disturbances. Breeds such as Meuse-Rhine-Yssel (**MRY**), SIM, Jersey (**JER**) and Dutch Friesian (**DFR**), had a significant breed effect with the estimates suggesting that they have a lower variance, a lower mean absolute residual and a smaller maximum residual. This suggests that these animals are either kept in environments with lower variability or disturbances, or that they cope better with the disturbances present, and as a result, keep their milk production more constant.

The daily deviations of the milk yield from the expected lactation curve formed the basis of 11 sensor features (category 4, Table 2). Our analysis suggests that, for these features, the MON, SRB and BSW do not deviate from the HOL with at most 4 out of 11 (36.4%) features different, similar to what we found for the features based on the Wood curve residuals in category 2. For SIM, MRY, BBL, JER and DFR cows, we found the most differences with up to 9 out of 11 (81.8%) features different. The model results show that these breeds have a less variable milk production in the environment they are kept in. Similar as for category 2, this suggests that either these cows are kept in less variable environments, or that they cope better with the disturbances present.

Eleven sensor features (category 5, Table 2) were quantified for the observed perturbations in DMY. In general, the non-HOL cows had less (major) perturbations, their perturbations lasted shorter both in the development and recovery phases, and they had lower milk losses. Accordingly, the combined breed and herd effects showed that almost all breeds had fewer or less severe perturbations than the HOL cows. This effect was largest for JER, BBL and MRY.

The last category of features (category 6, Table 2) were the resilience indicators developed by Poppe et al. (2020). Here, our results were similar to earlier work from Bonekamp et al. (2022), showing that 6 breeds (SIM, NRF, MRY, JER, GRO and DFR) had a significantly lower “LnVar” (i.e., natural logarithm of the variance of the residuals of the expected lactation curve) compared to HOL, which suggests that they have less daily fluctuations in milk yield either due to less variable environments or better coping with disturbances, or both. The breed effect on autocorrelation was significant for 5 of the breeds, but the direction of the effect differed across the breeds, with both positive (SRB, NRF and DFR) and negative estimates (MON and BSW) in comparison with the HOL cows. This suggests that the values for this resilience indicator are less consistent with other features and more difficult to interpret.

### 3.2 Herd comparison

#### 3.2.1 Dataset description

Subset B consisted of 361 herds (SEL50: *n* = 180; SEL99: *n* = 181) and 36,974 cows (*n* = 13,796 and *n* = 23,178 in SEL50 and SEL99, respectively). In SEL99, only 167 cows (0.65%) at 78 unique farms in the dataset were less than 7/8 HOL, whereas in SEL50, 783 animals (5.1%) at 112 unique farms had 7/8 HOL or more. SEL50 specifically consisted of following “main” breed, in which a cow was attributed to the breed with the largest breed part in this animal, without criteria for the actual breed proportion (Table 3): SIM: 27%, HOL: 25%, MRY: 18%, MON: 12%, JER: 4%, BSW: 4%, NRF: 3%, SRB: 2% DFR: 1%, GRO: 1%. For 3% of the animals, the main breed was recorded as ‘other’ or ‘unknown’. Table 3 also gives the average DMY for each of these categories based on the main breed. Cows with the highest proportion HOL and NRF had the highest DMY (24.3 and 24.6 kg respectively), which was lower than the average DMY of the purebred HOL cows in SEL99 (29.0 kg), and of the purebred HOL cows in Subset A (27.2 kg). The higher milk production in SEL99 compared to that of the HOL cows in Subset A suggests these SEL99 herds are highly specialised and intensive dairy farms with a deep focus on milk production. The cows in which the main breed part was GRO and JER had the lowest DMY (16.3 and 18.4 kg respectively). The cows assigned to the other breed categories all had average DMY between 20.4 and 23.0 kg per day. Other details on DMY, age at first calving and number of measurements are given in Supplementary Material (Supplementary Table S2).

**TABLE 3:** Average milk yield of cows with largest breed part in each breed in SEL50 and the contributions of breeds in SEL50.

Breed	DMY^a^ [kg]		Proportion of cows with main breed part in SEL50 [%]
	Mean	Std	
BSW	22.4	5.2	4
DFR	20.6	3.4	1
SIM	23.0	5.3	27
GRO	16.3	4.7	1
HOL	24.3	5.3	25
JER	18.4	4.2	4
MON	22.4	5.1	12
MRY	20.4	4.4	18
NR	24.6	4.7	3
UNK	23.0	4.9	3
SRB	23.0	5.4	2

^a^ DMY: average daily milk yield, BSW, brown swiss; DFR, dutch friesian; SIM, simmental fleckvieh; GRO, groninger whiteheaded; HOL, holstein friesian; JER, jersey; MON, Montbéliarde; MRY, Meuse-Rhine-Yssel, NRF, norwegian red, and UNK, other or unknown breeds; SRB, swedish red.

#### 3.2.2 Sensor features and resilience indicators in SEL50 vs. SEL99

Only a small proportion of the sensor feature variability could be explained by the DMY covariate or the percentage HOL in the herd (SEL), as demonstrated by the low *R* ^2^ of the models. For 39 out of the 40 features, we found a significant effect of the daily average milk yield (*p* < 0.00125). Only the number of sign changes of the residuals from the expected lactation curve (ExpAutoCorr) was not affected by the DMY scaling effect.

The SEL effect was small to very small but significant with a *p-*value lower than 0.00125 for 21 out of the 40 features. From these, 9 features had a direct link with the lactation curve shape and total milk production (category 1 and 3), as shown in Supplementary Table S3 of the Supplementary Material. The other 12 features for which the SEL variable was found significant mainly belonged to category 4 and 5 and represent the variability and dynamics of the residuals from the expected lactation curve and the perturbation characteristics (Table 4). More specifically, the residuals of the expected lactation curve showed a lower lag1 autocorrelation in SEL50 compared to SEL99. We found that there are in total less days in which the residuals of the expected lactation curve was negative, both per day and during 5 successive days. However, lactations in SEL50 had more individual days in which the DMY drops below 85% of the expected value. When considering the perturbation characteristics and dynamics, we see that for cows in SEL50 farms, there are a higher number of perturbations but that they last shorter both in the development and the recovery phase. The resulting milk losses are lower and the perturbations are less deep.

**TABLE 4:** Sensor features from category 4 and 5 for which a statistically significant (*p-*value < 0.00125) was found for the effect of SEL and the corresponding model interpretation.

Sensor feature^a^	Category	*p*-value	β1	Interpretation
ExpAClag1	6	0	−0.007	the residuals of the expected lactation curve in SEL50 lactations have a lower autocorrelation lag 1
ExpPercNegative	4	0	−0.004	there are less days with negative residuals in SEL50 compared to SEL99
ExpPercLower85	4	0	0.0036	there are more days in which the negative residuals drop below 85% of the expected milk production in SEL50 compared to SEL99
ExpNeg_5d	4	0	−0.0038	there are less periods in which the milk production drops below the expected for 5 successive days
PertNoMinor	5	0.0005	0.0033	there are more minor perturbations in SEL50 compared to SEL99
PertNoTotal	5	0	0.0053	there are more perturbations in total in SEL50 compared to SEL99
PertMinDaysRec	5	0	−0.0142	there are less days needed for recovery of minor perturbations in SEL50 than SEL99
PertMinDaysDev	5	0	−0.0092	there are less days needed for development of a minor perturbation in SEL50 than SEL99
PertMajMilkLoss	5	0.0006	−0.003	the total milk loss in minor perturbations is lower in SEL50 than in SEL99, despite correction for milk yield
PertMinMilkLoss	5	0	−0.0061	the total milk loss in major perturbations is lower in SEL50 than in SEL99, despite correction for milk yield
PertTotalLoss	5	0	−0.006	the total milk loss in all perturbations is lower in SEL50 than in SEL99, despite correction for milk yield
PertDeepest	5	0	0.0028	the largest perturbation has fewer milk loss at its deepest point compared in SEL50 compared to SEL99

^a^ For the detailed definitions of the sensor features, we refer to Supplementary Table S1 in the Supplementary Material of this manuscript.

Similar findings were derived from the sensor features and resilience indicators that were almost significant (*p*-values between 0.05 and 0.00125), as detailed in Supplementary Material (Supplementary Table S4). When all this is considered together, our analysis suggests that the cows in farms that belong to SEL50 have a higher variability in their milk production, but that this variability is mainly caused by short perturbations or single days in which the production drops. These differences suggest that either the average production environment differs across SEL50 and SEL99, with SEL50 having more disturbances that are generally milder or the cows on these farms deal differently with their environmental disturbances, or both.

#### 3.2.3 Sensor features and resilience indicators of purebred HOL cows in SEL50 and SEL99

To further investigate differences in sensor features and resilience indicators across the herds in SEL50 and SEL99, we compared the 782 purebred HOL cows in SEL50 with the 25,482 purebred HOL animals in SEL99. The average DMY of the former was 24.4 ± 5.4 kg, whereas this was 29.0 ± 5.3 kg for SEL99, demonstrating that either the HOL animals in SEL50 are less selected for milk production (and a genetic difference exists between the cows in each SEL despite they belong to the same breed), or they are kept in circumstances that are less optimal for these cows, or both.

To get a better view on differences in sensor features and resilience indicators across SEL50 and SEL99 herds, independent of the breed, the sensor features and resilience indicators from the purebred HOL cows in both groups of herds were compared (see Section 2.3.3.1; Eq. 3). The cows in SEL99 herds produced more milk (305-day yield: 8,676 kg vs. 7,262 kg for SEL50 and SEL99, respectively) and had a higher peak production (33.3 vs. 29.3 kg) which was reached 10 days later (DIM 63 vs. 53). The milk yield persistency was approximately equal. A similar result was found for the expected lactation curve dynamics (features of category 3). Features of category 2 calculated from the residuals of the Wood model, showed that the absolute residual of the Wood curve was slightly higher for SEL50 than for SEL99, indicating a bit more variability in the DMY in the herds of SEL50. Similar to what we found for the full dataset including all cows from all breeds, we found that SEL50 lactations have more minor perturbations and an equal number of major perturbations, but that both the development and recovery phases of these perturbations last shorter, with lengths of on average respectively 10 and 12 days for SEL50 vs. 13 and 15 days for SEL99. In conclusion, this analysis showed that purebred HOL cows in SEL50 have a higher variability in milk yield as captured with the LnVAR, RMSE and percentage negative residuals, but that the number and severity of their perturbations is lower. This suggests that the purebred HOL cows in the SEL50 herds are less affected by or less subject to very severe challenges or that the management and environment of these cows allows them to recover more quickly from a disturbance than HOL cows in SEL99 herds.

## 4 Discussion

### 4.1 Features based on high-frequency milk yield can capture response on challenges in the environment

When thinking of resilience, some non-HOL breeds are often perceived to be more robust, less vulnerable to diseases and more suited for more challenging environments in comparison with HOL cows (Rodríguez-Bermúdez et al., 2019; Windig & Hoving-Bolink, 2021). Good quality data demonstrating these differences have not been collected, as it requires quantification of all relevant aspects of the management, challenges, environment and performance characteristics of different farms (Dunne et al., 2019). An alternative is to measure the impact the environment has on the milk production dynamics. For example, Poppe et al. (2020) and Adriaens et al. (2020) suggested to use widely and automatically collected sensor data such as daily milk yield or activity measures to characterise the response of an animal on an environmental challenge. The idea is that, e.g., daily milk production strongly reflects how an animal copes with disturbances, for example, when a perturbation in milk production results from a health problem. Generally, fewer perturbations that last shorter and recover quickly are considered to reflect better resilience.

Our study used previously proposed sensor features and resilience indicators to quantify the milk yield dynamics of cows of different breeds and in herds with different breed proportions. We found that these features showed differences across our proposed groups in Subset A and B, suggesting that either the environments of herds in which these cows are kept consistently differ or the cows in the different groups differ in their response to environmental disturbances, or both. Unfortunately, the data did not allow to further test this explicitly.

More specifically, the sensor features and resilience indicators were assigned to six categories, each quantifying a different aspect of the milk yield dynamics. The lactation curve and expected lactation curve characteristics (category 1 and 3) represent the general milk production level over time, whereas category 2 and 4 quantify day-to-day variation, for example, reflecting response to differences in feeding or climate. The perturbations on the other hand (category 5) give more information on how cows respond to health challenges (e.g., mastitis) or other short or long-term environmental disturbances (e.g., heat stress). Hence, calculating and analysing these features separately has the advantage that the variability of the milk production for different situations can be obtained, all relevant for an aspect of the resilience of the animals. Besides a general view on how each animal individually thrives in its farm environment, a farmer can thus select and breed the animals that best suit their preference or the challenges posed on that specific farm. Additionally, this approach can be used to highlight aspects of the production environment and management within farm over time. For example, by monitoring the DMY based sensor features and resilience indicators, farm performance can be monitored, providing insight, e.g., in the effect of altered infection pressure or in management decisions such as changes in feed quality or grazing management.

Poor resilience shows differently in different cows, depending on their health history, age, management, nutrition, etc., and definition of one single indicator for resilience, e.g., based on a latent variable combining the features is deemed suboptimal. Hence, by quantifying each of the sensor features and resilience indicators separately, these variables can be used more intelligently than when they would be united in (a few) latent variables, for example, by performing principal component analysis or clustering. Keeping the features separate in the different categories allows to quantify the type of resilience that is best suited to the farmer’s preferences and facilitates tailoring the herd to a specific farm situation. Thus, a more black box approach would be only interesting when there would be a universal idea on what ‘resilience’ is and how to measure it. Given the lack of consensus in literature, and the fact that each environment poses different challenges to the animals and therefore requires cows that respond to these challenges in a different way, a combined approach in which all the variables are given similar weights is not considered optimal. Still, when a specific breeding goal is defined, a gold standard is available and the production environment is known, using latent variables with the different sensor features and resilience indicators combined is possible and potentially useful. The analysis and definitions presented in this study provide a good basis to perform these next steps.

We found that the scaling effect of milk production indeed affected almost all sensor features and resilience indicators. This was the only factor that we could explicitly include in the statistical analysis, and we chose for entering it in the regression as a linear covariate to keep consistency with previous work (Poppe et al., 2020; Bonekamp et al., 2022). The true impact of milk yield on the sensor feature values might differ across sensor features and resilience indicators.

The remaining sensor feature variability is probably caused by multiple, potentially also interacting factors, such as the difference in data quality, difference in model fit of the expected lactation curves, management factors, the specifics for each sensor feature calculation and their potential interactions. For example, when looking at the number of perturbations, a single long perturbation can coincide with multiple shorter ones with a different cause. Ben Abdelkrim et al. (2021) suggested to also model the nested perturbations (perturbations in perturbations) to overcome this problem, but this would increase the complexity of the analysis, which together with its computational cost was not feasible for our data.

### 4.2 Sensor features and resilience indicators differ in herds with high and low proportion of HOL

We quantified sensor features and resilience indicators on farms with a low and high percentage of HOL genetics in their herds. The expectation was that farms with HOL cows only have specialised management that is strongly focused on high levels of milk production in which disturbances are kept out as much as possible (control model, Ten Napel et al., 2006). As no breed criteria were applied to SEL50, we expected to find higher standard deviations for the sensor features and resilience indicators across the cows in SEL50. Although the high average DMY (29.0 kg) in SEL99 indeed suggests that these farms are highly specialised to achieve the best production, the standard deviations of the sensor features and resilience indicators in SEL99 was as large as in SEL50.

Besides a scaling effect for milk yield, we found that many sensor features and resilience indicators differed across SEL50 and SEL99, suggesting that 1) the environment; 2) challenges the cows are subject to; and 3) the way cows react to these challenges vary across farms with high and low percentage HOL genetics. More specifically, we found that in general the milk production on the SEL50 farms was more variable, but that once it goes critically wrong, the SEL99 cows had more severe (longer, more milk loss) perturbations than the cows in SEL50, even after correction for differences in milk yield levels. Resilience quantified as deviation from a curve is only expressed in the presence of disturbances. The more successful a farm is in keeping out disturbances, the fewer perturbations will be visible. As cows are therefore relatively unprepared for disturbances, once the cows are challenged, the perturbation will likely be larger and longer. The results suggest thus that SEL99 herds offer a more controlled production environment than SEL50 herds (farmers allowing cows to adapt; ‘adaptation model’, Ten Napel et al., 2006). Besides a difference in environment, this can also be due to the fact that cows in SEL50 are less “challenged” metabolically, and thus have more spare capacity to deal with their environmental disturbances. Pinpointing the exact causes, however, is not feasible with the current data, nor do we expect that a single causality can be isolated to explain these effects.

Part of the differences in farm environment is also reflected by the comparison of the purebred HOL cows of SEL50 and SEL99. Despite that these cows have a similar genetic background, both milk yield, and variability and perturbation-related features differed. In theory, several explanations can be put forward for these differences: 1) they originate from differences in the management, e.g., feed quality; 2) the cows were submitted to different breeding strategies, for example, less focussed on milk production and more on health, resulting in altered genetic backgrounds of the cows despite that they belong to the same breed; 3) these cows are challenged less in terms of production, allowing them to have a different energy partitioning to deal with the imposed disturbances, resulting in a quicker recovery upon a challenge. In practice, it is unlikely that the 78 SEL99 and 112 SEL50 farms differ substantially and consistently between groups in their breeding strategy, as all data originate from farms with an automated milking system in the Netherlands. Hence, differences in management and controlling production conditions may have a bigger influence on the differences observed than genetic background. Unfortunately, further investigating or quantifying this was not possible with the current dataset.

### 4.3 Sensor features and resilience indicators differ across breeds

The HOL breed is globally the most used breed on intensive dairy farms (Oltenacu & Broom, 2010), as these cows are known for their high milk yields. With a changing climate, more interest in sustainable production, circular farming and diversification of food production systems, there is a higher need for cows that can thrive in more diverse environments and cope with low feed quality. Using alternative breeds with different characteristics could help meeting this need (Rodríguez-Bermúdez et al., 2019). The non-HOL cows in our study all had a lower production compared to the HOL cows. Our results showed that, after considering the scaling effect of milk production on the sensor feature values, differences across the different breeds exist, with non-HOL breeds having less variability in milk yield, and fewer severe perturbations. This can result from 1) a difference in their production environment (e.g., these cows are kept in herds with lower environmental variability, cows are better prepared for common disturbances or are less challenged metabolically), 2) a breed difference, in which the non-HOL cows cope better with the disturbances present, and as a result, keep their milk production more constant; 3) a combination of both. It is plausible that non-HOL purebred cows are kept on herds for which the management environment is closer to the SEL50 herds than the SEL99 herds. Therefore, the probability that the differences found are solely the result from a less variable production environment, is small. Hence, by quantifying these differences with the sensor features and resilience indicators, opportunities arise to better understand differences between animals and find the best animal-in-environment herd.

For most breeds, the results for the expected lactation curve residuals and perturbation sensor features were consistent. Nonetheless, it is interesting to see that in some cases the sensor features in either category differ, suggesting that these indeed quantify different aspects of the disturbances in a production environment or a cow’s response thereon. Summarising lactation dynamics in a single trait ignores the true variability in the possible responses of cows to challenges. For example, for SIM cows, 81% of the sensor features of the fourth category (expected lactation residuals) differed from the HOL, whereas only 27% of the perturbation features were significant. This suggests that the way a cow reacts to a disturbance, e.g., by showing shorter deviations and quicker recovery vs. having longer milder or more severe perturbations, can give different information on how animals cope with the challenges their environment poses.

Accordingly, the choice for using non-HOL breeds for improving resilience largely depends on the farm context and what aspect of resilience farmers wish to improve in their herd. It can be that some breeds show a better resilience when focussing on the residuals of the expected milk yield curve (e.g., SIM cows), while other breeds show a better resilience when considering the perturbations (e.g., JER cows). These differences offer opportunities to select the breed according to the specific environment and preferences of the farmers. It is important to keep in mind that the favourable combination of breed and herd effects on milk yield variability as a proxy of resilience were in this study always associated with a lower milk production level.

### 4.4 Study limitations, implications and future work

We studied milk yield sensor features and resilience indicators for a large number of lactations and compared them across different breeds and herds. The breed effects we estimated were confounded with the herd effect, as including this explicitly in the model was not possibly with our current dataset. As a result, we could only highlight differences between the groups knowing that they possibly result from herd and environment aspects alone, and the interpretation of the results should be made accordingly. Additionally, high-frequency milk meter data is typically only available for herds with an automated milking system, limiting its extrapolation possibilities to other farm types and restricting the conclusions to farms with a milking robot only. Still, we found substantial differences between farms when studying the sensor features and resilience indicators. In specific farm contexts, these features can be used as precision phenotypes for breeding and management decisions, for example, to move towards cows that are less dependent on favourable farming conditions. This allows diversification of dairy production according to different breeding and production goals, which supports the development towards a more diverse production landscape in which the sustainability is improved with minimal impact on the environment. Future research on data for which information on farm environment, management and breeding goals are available would be helpful to further clarify and understand the differences that we found in this study.

## 5 Conclusion

This study provides a first quantification into differences across milk yield sensor features and resilience indicators of cows at different herds and from different breeds. We found that these features are useful to quantify different aspects of how cows respond to environmental disturbances, and potentially also how farm environments differ in the type of challenges they pose to the cows. This information is valuable to support breeding and management decisions, and follow milk yield variability over time within farm. On farms with a low and high percentage HOL genetics respectively, we found that the cows in the former had more variable milk production but fewer severe perturbations. This suggests that the farms with less HOL have in general a more variable production environment, but once it goes wrong for the cows in the more specialised farms, the effect of the disturbance is larger. Comparing the purebred HOL cows thereby eliminating breed effects confirmed these findings. After correcting for a milk yield scaling effect, different aspects of the milk yield dynamics, milk yield variability and perturbation characteristics differed across breeds as compared to HOL cows. These differences can result from the herd environment being different, from the cows reacting differently to the disturbances or both. More specifically, our data suggest that the milk production of non-HOL cows and crossbred animals is more stable with less (severe) perturbations. The current data did not allow to further investigate which factors affect sensor features and dynamics most. Still, this study demonstrates the potential to use milk yield sensor features and resilience indicators as a tool to quantify how cows perform in their specific environment. The present variability suggests that it could be possible in the future to select within farm for features that best suit a farms’ breeding goal and environment in terms of milk yield resilience.

## Data Availability

The data analyzed in this study is subject to the following licenses/restrictions: These data are retrieved from a database from CRV, Arnhem, Netherlands and are owned by them. No data can be shared without their permission. Requests to access these datasets should be directed to CK, claudia.kamphuis@wur.nl.

## Supplementary material

SUPPLEMENTARY TABLE S1 Description of the sensor features and resilience indicators.

SUPPLEMENTARY TABLE S2 Overview of the data in the subset selected based on percentage Holstein-Friesian (HOL) genetics.

SUPPLEMENTARY TABLE S3 Results of the lactation curve shape related sensor features (category 1 and 3) that differ across herds in SEL50 and SEL99.

SUPPLEMENTARY TABLE S4 Results of the sensor features that differ across herds in SEL50 and SEL99 with an almost-significant *p*-value between 0.0125 and 0.05 (significant, but above significance threshold after Bonferroni correction for multiple testing).
